# Cardiovascular disease risk factors and socio-economic position of Africans in transition: the THUSA study

**Published:** 2007-07

**Authors:** HH Vorster, A Kruger, CS Venter, BM Margetts, UE Macintyre

**Affiliations:** Africa Unit for Transdisciplinary Health Research(AUTHeR), North-West University, Potchefstroomcampus, Potchefstroom; Africa Unit for Transdisciplinary Health Research(AUTHeR), North-West University, Potchefstroomcampus, Potchefstroom; Africa Unit for Transdisciplinary Health Research(AUTHeR), North-West University, Potchefstroomcampus, Potchefstroom; Africa Unit for Transdisciplinary Health Research(AUTHeR), North-West University, Potchefstroomcampus, Potchefstroom; Institute for Human Nutrition, University of Limpopo (Medunsa campus), Pretoria

## Abstract

**Summary:**

In many developing countries with advanced stages of the nutrition transition, the burden of coronary artery disease (CAD) has shifted from the rich to the poor. In South Africa, it is mainly the African population that is experiencing rapid urbanisation and the nutrition transition. It is not clear where the burden of CAD lies in this population group. We tried to answer this question by comparing CAD risk factors within African groups of different socio-economic positions (characterised by total household income and education level) that participated in the THUSA study from 1996 to 1998. The THUSA study was a cross-sectional population-based epidemiological study that examined the influence of urbanisation and related changes in lifestyle and eating patterns on health and disease risk. A total of 1 854 apparently healthy African volunteers were recruited from 37 randomly chosen sites in rural and urban areas of the North-West Province.

The results indicated that although the group with the highest socio-economic position had significantly lower serum glucose levels, systolic blood pressures, higher micronutrient intakes and fewer smokers, their sustained increases in total and saturated fat intakes and higher serum total and LDL cholesterol levels, as well as increased body mass indices in men suggested that at that point in time and possibly in the foreseeable future, the burden of CAD will be carried by those Africans with higher socio-economic positions.

## Summary

Coronary artery disease (CAD) as well as other non-communicable diseases (NCDs) are rapidly becoming major causes of death in the developing world.^1^,^2^ This is partly because of the successful control of infectious diseases and demographic changes, but also due to changing lifestyles and dietary patterns.^3^ The latter, known as the nutrition transition, has started at different stages in different countries.^3^ It can be expected that in the early stages of the transition, the economically active and richer members of the population will carry the highest risk of CAD and other NCDs. However, there are indications from Mexico,^4^ Brazil^5^ and Chili^6^ that as the nutrition transition progresses, the burden shifts to the poor.

Sub-Saharan Africa is the poorest region in the world. There have been opinions and reports^7^,^8^ that as long as Africa remains impoverished, it is unlikely that CAD will emerge as a significant health problem. By contrast, the INTERHEART Africa study,^9^ examining the impact of modifiable CAD risk factors on myocardial infarction concluded that although there were low numbers of myocardial infarction cases, especially outside South Africa, with growing urbanisation and economic development, the rates may increase and resemble those seen in South Africa.

There is little doubt that CAD is a major health problem in all population groups of South Africa. In 2000, CAD contributed 23, 41, 31 and 52% of the total age-standardised death rates in the African, white, coloured and Indian population groups, respectively.^10^ The lower percentage in the African group (who had the highest death rate) was related to the high prevalence of deaths from HIV/AIDS. Several studies have shown that urbanisation and the nutrition transition in South Africa is accompanied by an increase in the CAD risk factors in Africans.^11^,^12^ It is, however, not clear if this increase in CAD risk is related to urbanisation *per se* or whether socio-economic position influences the nutrition transition and related increase in CAD risk. In other words, at what stage of the transition are black South Africans? Is CAD risk a burden of the rich or the poor in this population? An answer to these questions will help to estimate the future burden of the disease in those Africans who survive the HIV/AIDS epidemic, and will give guidance for targeting preventive measures.

The purpose of this analysis of data from the THUSA (Transition and Health during Urbanisation of South Africans) study was to determine the interaction between socio-economic position and risk factors for CAD in the African population of the North-West Province.

## Methods

The THUSA study was designed to examine the influence of urbanisation on the health of South Africans.^12^

A statistical model was used to recruit 1 854 apparently healthy volunteers, aged 15 years and older from 37 randomly selected sites from rural and urban areas in the North-West Province from 1996 to 1998. Subjects were stratified into five levels of urbanisation based on area of residence and type of employment. The levels of urbanisation were those in deep rural areas, farm workers, those living in informal housing areas (squatter camps), those living in traditional black locations (urban), and westernised subjects (upper urban).

Pregnant and lactating women, subjects with any known diagnosed disease, those with oral body temperatures above 37°C, those using any form of chronic medication and inebriated subjects were excluded.

All measurements with appropriate validation and quality-control procedures have been published.^12^,^13^ Briefly, demographic information (including education level, household composition and total income from all sources), health histories, lifestyle data (eg, smoking habits, physical activity) as well as habitual dietary and alcohol intakes were obtained during individual interviews by trained, multilingual field workers in the language of the subject’s choice, using structured and validated questionnaires. Nutrient intakes were calculated with the Medical Research Council’s Foodfinder programme, based on the South African food composition tables.^14^ Anthropometric measurements were taken by a level two anthropometrist and post-graduate students. Blood pressures were measured by trained and experienced researchers after subjects were seated for at least 10 minutes.

Fasting blood samples were drawn from the vena cephalica by registered nursing sisters using a sterile butterfly infusion set and disposable syringes. Samples were centrifuged in the field using Universal 16R Hettich centrifuges with cooling facilities. Aliquotes of serum, plasma and cells were stored at −20°C in the field for a maximum of five days and then transferred to a freezer at −84°C in the laboratory in Potchefstroom. Biochemical analyses of serum and plasma were done in the physiology and nutrition laboratories in Potchefstroom (glucose, insulin, fibrinogen, etc) and the chemical pathology laboratory, University of Pretoria (serum proteins, enzymes, lipids, etc, using the discrete analyzer, Technicon DAX48 system).

The Potchefstroom (now North-West) University ethics committee approved the study (no 4M5-95). The cooperation of the North-West Department of Health and Social Services and the permission of the various communities and their leaders were obtained during the planning phase of the study. All subjects signed an informed consent form (illiterate subjects with a cross). Volunteers identified with hypertension, diabetes mellitus or anaemia were referred to their nearest health facility for treatment. The subjects’ travel expenses were paid and they received sandwiches and fruit juice after blood samples were taken.

The SPSS package (version 15.0, SPSS Inc) was used to analyse the data. Data not normally distributed were normalised by logarithmic transformations. Means and 95% confidence intervals of CAD risk and dietary factors were calculated according to urbanisation level, total household income level, education level, and having a job (yes or no) ‘at present’. The socio-economic characteristics of the smokers were obtained using the Crosstabs procedure. Significant differences between estimated means in the different groups of men and women were determined with analysis of variance using the GLM (general linear model) multivariate procedure. Univariate analyses of variance were used to further explore the influence of total household income on CAD risk factors.

## Results

## Effects of urbanisation on CAD risk factors

The effects of urbanisation on mean levels of selected risk factors for CAD are shown in Table 1 for men and Table 2 for women. In both men and women, a significant increase in total serum cholesterol, because of increases in LDL cholesterol, were observed in upper urban subjects. Women of the urban group also showed increased levels of LDL-C. Mean plasma fibrinogen levels, which is a major risk factor for coronary heart disease and especially stroke,^15^ were higher in the male farm workers and squatter camp dwellers and the highest in urban women.

**Table 1.** Mean (95% CI) Cardiovascular Disease Risk Factors In African Men In Transition

*Risk factor (means and 95% confidence interval)*	*Deep rural*	*Farm workers*	*Squatter camps*	*Urban*	*Upper urban*
Numbers (*n*)	175	100	119	207	54
Age (years)	42.4^ab^	36.1^c^	35.5^a^	37.6^b^	31.0^bc^
	40.1−44.7	33.1−39.1	32.7−38.2	35.5−39.6	26.9−35.1
*Serum lipids (mmol/l)*					
Total cholesterol	4.0^a^	4.0^b^	3.8^c^	4.0^d^	4.6^abcd^
	3.8-4.1	3.8−4.2	3.6−4.0	3.9−4.1	4.3−4.9
HDL cholesterol	1.2	1.2	1.2	1.2	1.2
	1.2−1.3	1.1−1.2	1.1−1.3	1.2−1.3	1.1−1.3
LDL cholesterol	2.4^a^	2.4^b^	2.2^c^	2.4^d^	2.9^abcd^
	2.2−2.5	2.2−2.5	2.0−2.3	2.2−2.5	2.7−3.2
Triglycerides	1.2	1.2	1.1^a^	1.2	1.4^a^
	1.1−1.3	1.0−1.3	0.9−1.2	1.1−1.3	1.2−1.6
Fasting serum glucose (mmol/l)	5.0^a^	4.7^b^	4.9^c^	4.9^d^	4.2^abcd^
	4.8−5.1	4.5−5.0	4.7−5.1	4.7−5.1	3.8−4.5
Plasma fibrinogen (g/l)	3.0^ab^	3.4^ac^	3.2^b^	3.1^c^	3.2
	2.8−3.1	3.2−3.6	3.0−3.4	2.9−3.2	2.9−3.4
Systolic blood pressure (mmHg)	125^ab^	125^cd^	131^ace^	128^bdf^	122^ef^
	122−127	121−127	128−134	126−131	117−126
Diastolic blood pressure (mmHg)	75^ab^	76^c^	80^ac^	77	79^b^
	74−77	73−78	76−82	76−79	76−82
Body mass index (kg/m^2^)	20.9^a^	20.5^b^	20.2^ce^	21.3^cd^	22.7^abde^
	20.3−21.4	19.8−21.3	19.6−20.9	20.6−21.8	21.8−23.7

^a,b,c,^… Means with the same symbol differ significantly (*p* ≤ 0.05; pairwise comparisons, GLM, ANOVA).

**Table 2.** Cardiovascular Disease Risk Factors In African Women In Transition

*Risk factor (means and 95% confidence interval)*	*Deep rural*	*Farm workers*	*Squatter camps*	*Urban*	*Upper urban*
Numbers (*n*)	257	139	153	265	92
Age (years)	39.8^ab^	37.0^c^	36.5^ad^	38.2^e^	30.4^bcde^
	38.1−41.5	34.7−39.3	34.3−38.7	36.5−39.9	27.5−33.3
*Serum lipids (mmol/l)*					
Total cholesterol	4.1^ab^	4.1^cd^	4.1^ef^	4.5^ace^	4.5^bdf^
	3.9−4.2	3.9−4.3	3.9−4.2	4.3-4.6	4.3−4.8
HDL cholesterol	1.2	1.2	1.1^a^	1.1	1.2^a^
	1.1−1.2	1.1−1.2	1.1−1.2	1.1−1.2	1.1−1.3
LDL cholesterol	2.5^ab^	2.5^cd^	2.5^ef^	2.9^ace^	2.8^bdf^
	2.4−2.7	2.4−2.7	2.4−2.7	2.8−3.0	2.6−3.0
Triglycerides	1.2	1.1^a^	1.1	1.2^a^	1.1
	1.1−1.2	1.0−1.2	1.0−1.2	1.2−1.3	1.0−1.3
Fasting serum glucose (mmol/l)	4.9^a^	4.7^b^	5.1^c^	4.7^d^	4.1^abcd^
	4.7−5.1	4.5−5.0	4.9−5.4	4.5−4.9	3.8−4.5
Plasma fibrinogen (g/l)	3.3^abcd^	3.6^a^	3.7^b^	3.8^c^	3.7^d^
	3.1−3.4	3.5−3.8	3.6−3.9	3.6−3.9	3.5−4.0
Systolic blood pressure (mmHg)	126^abc^	128^d^	130^ae^	130^bf^	117^cdef^
	123−128	124−131	127−133	127−133	112−121
Diastolic blood pressure (mmHg)	77.1^abch^	77^def^	81^adg^	81^behi^	73^cfgi^
	75−78	75−79	79−83	79−82	70−75
Body mass index (kg/m^2^)	25.8^a^	26.3^b^	26.5^c^	28.2^abc^	27.3
	24.9−26.6	25.1−27.4	25.4−27.5	27.4−29.0	25.9−28.7

^a,b,c,^… Means with the same symbol differ significantly (*p* ≤ 0.05; pairwise comparisons, GLM, ANOVA).

In both men and women, mean fasting serum glucose levels and blood pressures were lowest in the upper urban group. Body mass index steadily increased with urbanisation level, although in women the mean BMI of the upper urban group was not significantly higher than those of the other groups: the women from the urban group had the highest mean BMI but overweight was present in all levels of urbanisation in women. These differences in risk factors (total cholesterol and BMI) were unlikely to be due to age differences since in both men and women, the rural groups with the lowest levels were significantly older than some of the other groups, while the upper urban groups were the youngest.

## Effects of level of household income

Table 3 and 4 illustrate the differences in CAD risk factors in the different categories of total household income. In men, there was a gradual but significant increase in total serum cholesterol levels with increased income (from 3.9 to 4.6 mmol/l). This increase was a result of an increase in LDL-C levels (Table 3). The increase in mean total serum cholesterol levels in women from 4.2 to 4.5 mmol/l from the lowest to the highest income group (Table 4) was not significant. However, an analysis of variance (results not shown) with total cholesterol as dependent variable and controlling for gender, age, BMI, level of urbanisation and education, showed that the highest income category (R3 000 plus) had significantly higher total cholesterol levels (4.4 mmol/l) than all the other categories (varying from 4.1 to 4.2 mmol/l).

**Table 3.** Mean (95% CI) Cardiovascular Disease Risk Factors Of Men In Different Income Categories

*Risk factor (means and 95% confidence interval)*	*Total household income category (R/month)*					
	*R0-100*	*R101-500*	*R501-1000*	*R1001-2000*	*R2001-3000*	*R3000+*
Number (*n*)	101	194	159	111	40	50
Age (years)	35.1^a^	42.0^abcde^	37.3^b^	35.2^c^	33.9^d^	36.2^e^
	32.1−38.2	39.8−44.2	34.9−39.7	32.3−38.0	29.1−38.7	31.9−40.5
*Serum lipids (mmol/l)*						
Total cholesterol	3.9^a^	3.9^b^	3.9^c^	3.9^d^	4.2	4.6^abcd^
	3.7−4.1	3.8−4.1	3.8−4.1	3.7−4.1	3.9−4.5	4.3−4.8
HDL cholesterol	1.2	1.2	1.2	1.3	1.2	1.1
	1.1−1.3	1.1−1.3	1.1−1.2	1.2−1.3	1.2−1.3	1.2−1.3
LDL cholesterol	2.3^ab^	2.3^c^	2.3^d^	2.3^ef^	2.6^ae^	3.0^bcdf^
	2.1−2.4	2.2−2.5	2.2−2.5	2.1−2.4	2.3−2.9	2.7−3.2
Triglycerides	1.1a	1.1b	1.3c	1.1	1.3	1.4^abc^
	1.0−1.3	1.0-1.2	1.1−1.4	1.0−1.3	1.0−1.5	1.2−1.7
Serum glucose (mmol/l)	5.0	4.9	4.8	4.8	4.8	4.6
	4.7−5.2	4.7−5.1	4.6−5.0	4.5−5.0	4.4−5.3	4.3−5.0
Plasma fibrinogen (g/l)	3.2	3.2	3.1	3.1	2.9	3.3
	2.9−3.4	3.0−3.3	2.9−3.2	2.9−3.3	2.6-3.2	3.0−3.6
Systolic blood pressure (mmHg)	127	127	127	126	126	127
	124−130	125−130	125−130	123−129	120−131	122−132
Diastolic blood pressure (mmHg)	74^ab^	78^a^	77	77	76	80^b^
	72−77	76−79	75−79	75−79	73−80	77−84
Body mass index (kg/m^2^)	19.9^abcd^	20.9^ae^	21.1^bf^	20.8^g^	22.1^c^	22.8^defg^
	19.2−20-6	20.4−21.4	20.5−21.6	20.1−21.5	20.9−23.2	21.8−23.8

^a,b,c,^… Means with the same symbol differ significantly (*p* ≤ 0.05).

**Table 4.** Mean (95% CI) Cardiovascular Disease Risk Factors Of Women In Different Income Categories

*Risk factor (means and 95% confidence interval)*	*Total household income category (R/month)*					
	*R0-100*	*R101-500*	*R501-1000*	*R1001-2000*	*R2001-3000*	*R3000+*
Number (*n*)	161	299	237	133	33	43
Age (years)	36.6^ab^	40.7^acde^	38.7^fg^	30.7^bcf^	32.8^dg^	34.6^e^
	34.5−38.8	39.1−42.3	36.9−40.4	28.4−33.1	28.1−37.5	30.5−38.7
Serum lipids (mmol/l)						
Total cholesterol	4.2	4.2	4.3	4.2	4.3	4.5
	4.1−4.4	4.1−4.3	4.1−4.4	4.0−4.3	4.0−4.7	4.1−4.8
HDL cholesterol	1.2^a^	1.1	1.1	1.1^ab^	1.2	1.2^b^
	1.1−1.2	1.1−1.2	1.1−1.2	1.1−1.2	1.1−1.3	1.1−1.3
LDL cholesterol	2.6	2.6	2.7	2.6	2.8	2.7
	2.5−2.8	2.5−2.8	2.6−2.9	2.4−2.8	2.4−3.1	2.4−3.0
Triglycerides	1.1	1.2	1.2	1.1	1.1	1.0
	1.0−1.3	1.1−1.3	1.1−1.3	1.0−1.2	1.0−1.3	1.0−1.2
Serum glucose (mmol/l)	4.8	4.9^a^	4.8	4.6	4.7	4.3^a^
	4.5−5.1	4.7−5.1	4.6−5.0	4.3−4.9	4.1−5.3	3.8−4.9
Plasma fibrinogen (g/l)	3.6	3.6	3.6	3.6	3.7	3.7
	3.4−3.8	3.4−3.7	3.5−3.8	3.4−3.8	3.3−4.1	3.3−4.0
Systolic blood pressure (mmHg)	129^ab^	129^cde^	128^fg^	122^acf^	122^d^	119^beg^
	125−132	127−132	126−131	119−126	114−129	113−126
Diastolic blood pressure (mmHg)	78^a^	79^b^	79^cd^	76^c^	79^e^	73^abde^
	76−80	77−80	78−81	74−79	75−84	69−77
Body mass index (kg/m^2^)	25.8^ab^	26.1^cd^	27.4^ac^	28.1^bd^	28.3	27.8
	24.8−26.9	25.3−26.9	26.5−28.2	26.9−29.2	25.9−30.6	25.8−29.8

^a,b,c,^… Means with the same symbol differ significantly (*p* ≤ 0.05).

Both systolic and diastolic blood pressures of women in the higher income groups were significantly lower than those in the lower income groups. In men, no changes in mean systolic pressures were observed, but mean diastolic pressure of the highest income group was significantly increased compared to that in the lowest income group. The BMI of men and women increased with increasing income levels. In women, however, the mean BMIs of those in the two highest income levels were not significantly higher than those in the lower income groups. Again, it is unlikely that mean age was responsible for the observed differences, since the oldest men and women were in the second lowest income level.

## Effects of level of education

Table 5 shows the mean (95% CI) levels of some of the CAD risk factors of subjects grouped into five different education levels. Subject groups with none or lower education levels (groups 1 and 2) were significantly older. Despite these age differences, the table shows that serum cholesterol levels increased significantly with increasing education. Again, this was because of increases in LDL cholesterol (data not shown). Significantly lower mean serum glucose levels and systolic blood pressures were observed in both men and women with increased education levels. The mean BMI of the men, but not of the women, increased significantly with increased education level.

**Table 5.** Mean (95% CI) Of Selected CVD Risk Factors Of Men And Women With Different Education Levels

*Risk factor*	*Gender*	Education level*				
		*1*	*2*	*3*	*4*	*5*
Number (*n*)	Men	162	184	130	123	50
	Women	174	311	195	160	63
Age (years)	Men	49.9^abcd^	40.8^aefg^	30.0^behi^	25.8^cfhj^	35.1^dgij^
		47.9−51.9	38.9−42.6	27.8−32.2	23.5−28.1	31.5−38.7
	Women	47.2^abcd^	42.6^aefg^	31.1^befhi^	26.0^cgij^	32.7^dhj^
		45.5−49.0	41.3−44.0	29.5−32.8	24.1−27.8	29.8−35.6
Total serum cholesterol (mmol/l)	Men	4.1^abc^	4.0^def^	3.7^adg^	3.7^beh^	4.8^cfgh^
		3.9−4.2	3.9−4.2	3.6−3.9	3.6−3.9	4.5−5.1
	Women	4.3^ab^	4.4^cd^	4.0^ac^	3.9^be^	4.5^de^
		4.2−4.5	4.3−4.5	3.9−4.2	3.8−4.1	4.3−4.8
Fasting serum glucose (mmol/l)	Men	5.1^abc^	4.8^a^	4.9^d^	4.6^b^	4.5^cd^
		4.9−5.3	4.6−5.0	4.7−5.1	4.4−4.9	4.1−4.8
	Women	4.8^a^	5.1^bcd^	4.6^b^	4.6^c^	4.2^ad^
		4.6−5.1	4.9−5.2	4.4−4.9	4.3−4.9	3.8−4.6
Systolic blood pressure (mmHg)	Men	131^abcd^	127^ae^	125^b^	123^ce^	124^d^
		129−134	125−130	123−128	120−126	119−128
	Women	138^abcd^	131^aefg^	124^behi^	117^cfh^	118^dgi^
		135−141	128−133	121−127	113−120	113−123
Diastolic blood pressure (mmHg)	Men	79^ab^	77^c^	76^ad^	75^be^	82^cde^
		77−81	75−79	74−78	73−77	79−85
	Women	82^abc^	80^def^	77^adg^	73^beg^	75^cf^
		80−84	79−81	75−79	71−75	72−79
Body mass index (kg/m^2^)	Men	20.8^a^	20.8^b^	20.8^c^	20.6^d^	23.8^abcd^
		20.3−21.4	20.2−21.3	20.1−21.4	20.0−21.3	22.8−24.8
	Women	26.9	27.5^a^	27.4	26.0^a^	26.9
		25.9−27.9	26.8−28.3	25.4−27.3	25.0−27.1	25.3−28.6

*Education level: 1 5 none; 2 5 < grade 8; 3 5 grades 8−10 with/without trade; 4 5 grades 11−12 with/without trade; 5 5 ≥ grade 12 plus academic training. ^a,b,c,^… Means with the same symbol differ significantly (*p* ≤ 0.05).

## Effects of smoking

Although the amount of pipe tobacco and the number of cigarettes smoked per day, as well as snuff taking (mainly women) were measured,^12^ for the purpose of this analysis, subjects were divided into ‘present’ smokers and non-smokers (never-smokers). Table 6 gives the percentages of smokers per urbanisation, household income and education categories, as well as the percentage smokers in those with and without jobs ‘at the moment’. The table shows that whereas 57% of all men smoked, only 17% of women reported the habit. Men in the upper level of urbanisation smoked less than those in the other groups. Income and level of education had larger impacts on smoking in women than in men. Of men with jobs ‘at pesent’, 63% smoked whereas 50.4% of those without jobs smoked. The corresponding figures for women were 16.8 and 17.4%.

**Table 6.** Percentage Smokers Within Group According To Level Of Urbanisation, Income, Employment And Education Level

*Categories*	*% smokers in category*	
	*Men*	*Women*
Urbanisation level		
Deep rural	49.5	20.5
Commercial farms	65.8	27.6
Informal settlements	64.2	21.0
Urban	60.8	11.7
Upper urban	36.0	0.9
Income level (R/month)		
R0−100	47.4	23.2
R101−500	68.6	19.8
R501−1000	52.2	20.8
R1001−2000	57.6	5.5
R2001−3000	46.0	0.0
R3000+	51.6	1.9
Employment		
Job ‘at moment’	63.0	16.8
No job ‘at moment’	50.4	17.4
Education level		
None	66	31
Less than Grade 8	66	23
Grade 8−10 with/without trade	49	12
Grade 11−12 with/without trade	43	4
Grade 12 plus academic	46	0
Total group	57	17

## Effects of nutrient intakes

The mean intakes of some nutrients selected because of their known effects on CAD risk (eg, macronutrients) and because they illustrate diet quality (eg, micronutrients) are reported in Tables 7 and 8 for men and women, respectively, stratified into the different income groups. In men (Table 7) from the lowest income category to higher income levels, the largest increases in energy, animal-derived protein, total fat, saturated fat and dietary cholesterol were seen in the middle income categories: in the highest income category, the increases were either smaller or did not differ from the lowest category.

**Table 7.** Mean (95% CI) Of Selected Nutrient Intakes Of African Men Per Income Category

*Nutrient*	*Total household income category (R/month)*					
	*R0−100*	*R101−500*	*R501−1000*	*R1001−2000*	*R2001−3000*	*R3000+*
Total energy from food (MJ)	8.6^a^	8.6^b^	9.0^c^	9.8^abc^	9.6	8.9
	8.0−9.3	8.2−9.1	8.4−9.5	9.2−10.5	8.5−10.7	7.9−9.8
Total protein (g)	66^ab^	62^cd^	63	74^ac^	73^bd^	69
	62−71	59−65	60−67	69−78	65−81	62−76
Animal-derived protein (g)	30^abc^	26^adef^	25^bghi^	34^dg^	36^ceh^	35^fi^
	27−33	24−28	22−27	31−36	32−41	31−39
Total fat (g)	60^abc^	52^adef^	56^gh^	67^bdg^	69^ceh^	63^f^
	55−65	48−56	52−60	62−72	61−77	56−71
Saturated fat (g)	18^a^	16^abcd^	17^efg^	20^be^	21^cf^	20^dg^
	17−20	15−17	16−19	18−22	18−24	18−23
Cholesterol (mg)	338^ab^	295^cde^	320^fg^	419^acf^	443^bdg^	370^e^
	294−381	264−327	285−354	377−461	374−512	308−432
P/S ratio	0.8^ab^	0.9^ac^	1.0^bd^	0.9^e^	0.8	0.8^cde^
	0.8−0.9	0.9−1.0	0.9−1.0	0.9−1.0	0.7−1.0	0.7−0.9
Total carbohydrate (g)	316^a^	342	350	367^a^	349	321
	286−347	320−363	326−374	339−396	301−397	278−364
Dietary fibre (g)	19	17^a^	18	20^a^	19	18
	17−21	15−18	17−20	18−21	16−22	16−21
Calcium (mg)	448	463	451	486	448	455
	395−500	426−501	410−493	436−536	365−532	380−529
Iron (mg)	9.6^a^	8.1^abcd^	8.8^e^	10.4^be^	9.7^c^	9.7^d^
	8.7−10.4	7.5−8.7	8.1−9.5	9.6−11.3	8.3−11.0	8.5−10.9
Vitamin A (RE)	702^abc^	554^ade^	638^fg^	928^bdfh^	980^cegi^	723^hi^
	584−820	468−639	544−732	815−1040	793−1168	555−891
Ascorbic acid (mg)	39^abc^	27^ade^	29^bfg^	34^hi^	50^dfh^	51^cegi^
	33−46	22−31	24−35	28−40	39−60	42−60

^a,b,c,^ Means with the same symbol differ significantly (*p* ≤ 0.05). P/S: ratio of polyunsaturated to saturated fat.

**Table 8.** Mean (95% CI) Of Selected Nutrient Intakes Of African Women Per Income Category

*Nutrient*	*Total household income category (R/month)*					
	*R0−100*	*R101−500*	*R501−1000*	*R1001−2000*	*R2001−3000*	*R3000+*
Total energy from food (MJ)	7.8^.a^	7.9	7.9	8.3	8.8	8.7^a^
	7.3−8.3	7.6−8.3	7.5−8.2	7.7−8.8	7.8−9.8	7.8−9.6
Total protein (g)	57^ab^	55^cde^	55^fgh^	61^cfi^	69^adg^	72^behi^
	54−61	53−58	53−58	58−65	62−76	66−79
Animal-derived protein (g)	27^abcde^	23^afgh^	24^bijk^	31^cfilm^	40^dgjl^	44^ehkm^
	24−29	22−25	22−25	29−34	35−45	40−48
Total fat (g)	53^abc^	50^def^	52^ghi^	59^adgj^	68^beh^	77^cfij^
	49−57	47−53	49−55	55−64	60−77	70−85
Saturated fat (g)	17^abc^	16^def^	16^ghi^	19^adgjk^	23^behj^	26^cfik^
	15−18	15−17	15−17	17−20	20−26	23−28
Cholesterol (mg)	260^abc^	263^def^	283^g^	309^ad^	362^beg^	339^cf^
	229−290	241−286	257−308	275−342	294−430	280−399
P/S ratio	0.9^abc^	0.9^def^	0.9^ghi^	0.8^adgj^	0.7^beh^	0.7^cfij^
	0.8−1.0	0.9−0.9	0.9−1.0	0.8−0.9	0.6−0.8	0.6−0.8
Total carbohydrate (g)	284	305	293	298	303	273
	264−304	290−320	276−310	276−320	258−347	234−312
Dietary fibre (g)	16^a^	16^b^	16^c^	17	17	19^abc^
	15−17	15−17	15−17	16−19	15−20	17−22
Calcium (mg)	372^abc^	393^de^	387^fgh^	437^afi^	502^bdg^	572^cehi^
	336−408	366−419	357−416	398−477	423−582	502−641
Iron (mg)	8.2^abc^	8.0^def^	8.2^ghi^	9.3^adgj^	10.1^beh^	10.9^cfij^
	7.6−8.8	7.6−8.5	7.7−8.7	8.6−10.0	8.8−11.5	9.7−12.0
Vitamin A (RE)	772^abcde^	628^afgh^	628^bijk^	949^cfil^	1131^dgj^	1233^ehkl^
	665−879	550−707	540−716	832−1067	895−1367	1026−1440
Ascorbic acid (mg)	41^abcd^	28^aefg^	32^bhij^	47^ehkl^	72^cfik^	73^dgjl^
	35−47	24−32	27−37	40−54	59−86	62−85

^a,b,c,^ Means with the same symbol differ significantly (*p* ≤ 0.05). P/S: ratio of polyunsaturated to saturated fat.

Total carbohydrate and dietary fibre intakes were similar in all male groups, but the mean intakes of several micronutrients (eg, iron, vitamin A and ascorbic acid) indicative of dietary quality improved with increasing income levels. In women, the increases in total energy, protein, animal-derived protein, fat, saturated fat and dietary cholesterol were also seen in those with the highest income. The increases in dietary fibre intake, calcium, iron, vitamin A and ascorbic acid mirrored the increased income categories, and reflect a more varied diet with better micronutrient densities.

## Discussion

The purpose of this analysis of the THUSA data was to examine the relationship between socio-economic position (as reflected by household income and level of education) and CAD risk factors in Africans in transition, to determine if the reported increased risk of CAD with urbanisation^11^,^12^ is a burden carried by the rich or the poor. The relationships between social position and health in South Africa are complex^16^ because of historical and political reasons. However, urbanisation with accompanied acculturation, modernisation or westernisation is at present mainly taking place in the black population. The major changes in political structures in 1994 stimulated a rapid acceleration of urbanisation in this group, which makes a comparison of rural and urban blacks from 1996 to 1998 valid to determine the direction of the relationship between socio-economic position and CAD risk.

Urbanisation is of course linked with economic development and education level: which are the main reasons why people move from underdeveloped rural areas with limited job and education opportunities to more developed urban areas. But an improved social position is not necessarily an outcome of urbanisation. The urban slums throughout the developing world and, in South Africa in particular, the informal housing areas or squatter camps which were one of the groups examined in the THUSA study, attest to this.

The results of this analysis showed that with urbanisation, increasing income and increased education, there were marked and sustained increases in some CAD risk factors, notably total serum and LDL cholesterol levels in men and women, and BMI in men. The exception was that the increases in total cholesterol with increased income in women were not significant. The presence of overweight and obesity in women of all urbanisation, income and education categories may explain this observation. But the results also showed that those in the upper urban group, those with the highest income level (R3 000 plus per month) and those with the highest education level (Grade 12 plus after-school academic training), mostly had lower serum glucose levels, lower systolic blood pressures, and in women, lower BMIs. There may have been a number of reasons for this.

Firstly, it could mean that those of the highest social position had better access to healthcare. The THUSA study only recruited apparently healthy volunteers without diagnosed diseases, and found that in the rural and informal housing areas, many subjects who wanted to participate had undiagnosed hypertension or diabetes mellitus.^12^ The sustained increases in serum cholesterol levels (which is not often measured in Africans) support this argument.

The second possible reason is that the health transition in Africans is indeed at a stage where the burden of CAD and other NCDs is carried by those with a lower socio-economic position. However, the reported dietary changes during urbanisation of these subjects,^17^ as well as the nutrient intakes per income category, shown in Tables 7 and 8, do not support this argument. The increases in total and animal-derived protein, total and saturated fats as well as dietary cholesterol, and the decreases in the polyunsaturated/saturated fat ratios shown in Tables 7 and 8 were sustained in subjects with the highest incomes. These changes are all associated with CAD risk.^18^

With urbanisation^17^ and also with increases in household income (Tables 7, 8), micronutrient intakes also improved. Although mean levels did not reach recommended levels in all instances (eg, maximum mean calcium intakes of men and women were 448 and 502 mg/day, whereas the USA and Canada’s recommended intake is 800 mg/day^17^), it is conceivable that the higher intakes of the anti-oxidant micronutrients with increased household income could protect against some NCDs and CAD.

The high percentage of smokers among the men within the different categories is of concern. There are some indications that in the upper urban, highest income, and higher education level groups, fewer people smoked. Smoking of cigarettes is accepted as a major risk factor for CAD^2^ and the above pattern suggests that CAD may become a burden of the poor in the future. The results also indicated that overweight and obesity among women is becoming a problem among the rich and the poor in this population.

## Conclusion and recommendation

Although there were indications of lower CAD risk factors in the highest socio-economic group of this sample of Africans in transition, such as increased micronutrient intakes, lower percentages of smokers, lower serum glucose levels and lower systolic blood pressures, there were also indications that among women, overweight and obesity, and some of the risk factors of non-communicable diseases were present in both the rich and the poor. However, the sustained increases in total and saturated fat intakes, the higher total serum and LDL cholesterol levels, as well as the increased BMI of men in the highest socio-economic group showed that in the period from 1996 to 1998 and possibly in the foreseeable future, the CAD burden during the health transition will be heavier in those with a higher socio-economic position.

It is recommended that in addition to the present focus to prevent HIV infection, Africans should be informed about the CAD risk of their changing lifestyles. Special attention should be given to aggressively promote prudent, low-fat but micronutrient-rich diets, and to inform the total population about the health consequences of overweight, obesity and smoking.
